# Should Cancer Survivors Fear Radiation-Induced Sarcomas?

**DOI:** 10.1080/13577149778425

**Published:** 1997-03

**Authors:** Malcolm Feigen

**Affiliations:** The Radiotherapy Centre, Austin & Repatriation Medical Centre Repatriation Campus Locked Bag 1 Heidelberg West Victoria 3081 Australia

## Abstract

*Purpose/Results*. Ionizing radiation is carcinogenic and the induction of a second malignancy is a serious potential long-term complication of radiotherapy. The incidence of radiation-induced sarcomas was evaluated from many large epidemiological surveys of long-term cancer survivors reported in the literature over the past 30 years and only one case was found for every 1000 patients irradiated.

*Discussion*. Although greater numbers of cancer patients are receiving radical radiotherapy and surviving free of disease for longer intervals, cases of radiation-induced sarcomas are rare and should not deter patients from accepting radiotherapy as treatment for curable cancers. With improvements in the administration of radiotherapy over the past two decades which are resulting in less damage to bone and soft tissues, it is likely that fewer cases of this condition will be seen in the future. If these sarcomas are diagnosed early, long-term survival can be achieved with surgical excision and possibly re-irradiation, as occurs in other types of sarcomas.


*Sarcoma* (1997) **1**, 5–15

**REVIEW**

### Should cancer survivors fear radiation-induced sarcomas?

MALCOLM FEIGEN

*The Radiotherapy Centre, Austin & Repatriation Medical Centre, Victoria, Australia*

**Abstract**

*Purpose/Results.* Ionizing radiation is carcinogenic and the induction of a second malignancy is a serious potential long-term complication of radiotherapy. The incidence of radiation-induced sarcomas was evaluated from many large epidemiological surveys of long-term cancer survivors reported in the literature over the past 30 years and only one case was found for every 1000 patients irradiated.

*Discussion.* Although greater numbers of cancer patients are receiving radical radiotherapy and surviving free of disease for longer intervals, cases of radiation-induced sarcomas are rare and should not deter patients from accepting radiotherapy as treatment for curable cancers. With improvements in the administration of radiotherapy over the past two decades which are resulting in less damage to bone and soft tissues, it is likely that fewer cases of this condition will be seen in the future. If these sarcomas are diagnosed early, long-term survival can be achieved with surgical excision and possibly re-irradiation, as occurs in other types of sarcomas.

**Key words**: *radiation-induced sarcoma, bone sarcoma, soft tissue sarcoma.*

### Introduction

Advances in cancer treatments are producing increasing numbers of healthy long-term survivors and, as a consequence, the delayed complications of anti-cancer therapies are assuming a greater significance. Radiation has been recognized both as a therapeutic agent capable of curing localized malignancies and as a mutagen which may induce new cancers. It is just under 100 years since the first cases of radiation-induced tumours were recognized, in the first decade following the discovery of X-rays by Röntgen in 1895, when pioneer radiographers were reported with squamous cell carcinomas on exposed hands[1] and with leukaemias. A soft tissue sarcoma induced by radiation was described in 1904 by Perthes[2] and, in the 1920s, bone sarcomas were noted following irradiation for tuberculous arthritis and radium exposure of workers painting watch dials.[3,4] Cancer patients may develop second neoplasms spontaneously or as a consequence of the same genetic or environmental factors which led to the development of their first tumour, and the role of radiation can be difficult to establish since no specific histological or biochemical markers have yet been identified. However, specific cases may be attributed to radiotherapy when they develop in normal tissues that have been previously irradiated, following a reasonable induction interval, particularly if the histology is unlike the usual tumours of the region.

Sarcomas can be categorized as radiation induced if they meet the following criteria, adapted from the original prerequisites established by Cahan *et al.*:[5]

(1) the sarcoma must develop within the boundaries of a previously irradiated area;
(2) a relatively long asymptomatic latent period (at least 4 years) must have elapsed;
(3) the sarcoma must have a different histology from the original lesion;
(4) the sarcoma must be histologically confirmed.

Bone and soft tissue sarcomas induced by therapeutic irradiation are frequently advanced by the time they are diagnosed and are usually incurable. With current trends to use megavoltage radiotherapy for organ conservation as an alternative to surgery, often in combination with intensive chemotherapy protocols, in growing numbers of cancer patients, it is important to establish how commonly these cases occur and find ways to diagnose them early when they are amenable to the curative treatments that are applied for primary sarcomas. The literature was reviewed to define the incidence of cases of bone and soft tissue sarcomas which have

Correspondence to: M. Feigen, The Radiotherapy Centre, Austin & Repatriation Medical Centre, Repatriation Campus, Locked Bag 1, Heidelberg West, Victoria 3081, Australia. Tel: +61 3 9496 2800; Fax: +61 3 9496 2826; E-mail: feigenm@ozemail.com.au.

1357-714X/97/010005-11 ©1997 Journals Oxford Ltd developed within radiation fields of patients who were treated by radiotherapy and survived beyond a reasonable latent period to allow time for the development of a radiation-induced sarcoma.

### Discussion

### *Radiation-induced sarcomas*

A literature review of 344 sarcomas following irradiation undertaken by Robinson and colleagues[6] in 1988 found that the median latent interval was 11 years and the median survival was 12 months, with only 11% of patients alive after 5 years. Most patients were diagnosed at an advanced stage with sarcomas that were high grade and usually unresectable, often developing metastases and unresponsive to chemotherapy. Histologically, the largest group comprised osteosarcomas, followed by fibrosarcomas, malignant fibrous histiocytomas, angiosarcomas, chondrosarcomas and others. The commonest primary was breast cancer, then gynaecological cancers and retinoblastoma.

The range of radiation doses found to induce sarcomas is wide and tends to be higher for megavoltage than orthovoltage (DXR) treatments. These modalities have different photon absorption patterns which have led to the phasing out of orthovoltage radiotherapy for the majority of cancer patients over the past 40 years. Bone tumours and sarcomas in cutaneous and subcutaneous tissues are more common after orthovoltage therapy,[7] which delivers to bone approximately twice the dose that is absorbed by adjacent soft tissues. Some reviews have noted a dose–response relationship for radiation doses in excess of 10 Gy,[8,9] below which very few cases of radiation-induced sarcomas have been reported. It has not been established whether children are at higher risks than adults, allowing for the longer time interval for them to develop sarcomas.[10]

Population-based studies of radiation-induced sarcomas have uniformly found very low incidence rates. Figures reported from large cancer centres range from 0.03% and 0.38% of all 5-year survivors[8,11,12] to 0.27% of all 10-year survivors.[13] Between 1% and 3% of all sarcomas have been associated with prior irradiation to the sarcoma site, and soft tissue sarcomas are three times as common as bone sarcomas.[7,14,15]

A number of familial conditions have been linked to multiple cancers including sarcomas, and it is likely that radiation will increase the incidence of sarcomas occurring in these patients. This has been long appreciated in retinoblastoma patients,[16] where the genetic cases with bilateral tumours have a high rate of sarcomas within the radiation port as well as at more peripheral sites. Neurofibromatosis[17] and the Li-Fraumeni syndrome[18] are two other autosomal dominant genetic conditions where sarcomas may develop in the absence of radiotherapy, and the sarcoma classified as radiation induced when this is but a secondary factor.

Other factors which have been associated with the development of sarcomas in large numbers of case reports include excessive radiation damage to skin, bone and soft tissue, and the contributory effects of chemotherapy. The majority of cases have been reported following orthovoltage radiotherapy, where frequently higher daily dose fractions were given than is acceptable in current radiotherapy practice. Patients were often exposed to multiple courses of radiotherapy to the sarcoma site and total doses were commonly far in excess of what is now regarded as the radiation tolerance dose for the tissue. It is probable that some chemotherapy regimens stimulate sarcoma induction,[19–21] and in children at least, alkylating agents may double the risk.[9] For many cancers the total doses of adjuvant chemotherapy have been reduced in recent years following evidence that less intensive regimens are equally effective.

### *Retinoblastomas*

Retinoblastoma is a cancer affecting young children which can be cured by radiotherapy. Inherited in around 40% of cases, genetic cases are usually bilateral and follow an autosomal dominant pattern of inheritance. There is a strong association with sarcomas, which occur both within radiation portals and at other sites, frequently in patients who have not been given radiotherapy.

The largest study of 1603 long-term retinoblastoma survivors from New York and Boston hospitals treated from 1914 to 1984 by Eng and colleagues[22] found that 6.6% died from second tumours after a median follow-up of 17 years from radiotherapy, resulting in a cumulative probability of death from second primary neoplasms of 26% at 40 years following diagnosis for patients with bilateral disease. However, this report included cases other than sarcomas, and an unspecified number occurred outside the field of irradiation. Second tumours are not always fatal, and many are cured by aggressive treatment.[23]

Radiotherapy has a well-established role in the treatment of retinoblastomas, curing early cases with the preservation of useful vision. Bone and soft tissue sarcomas may develop irrespective of whether the patient has received radiotherapy or not, and the recommended optimal radiation dose is now half that previously given. As a further benefit, it has been noted that prophylactic retinal radiotherapy can significantly reduce the incidence of contralateral retinoblastomas in familial cases, using a radiotherapy technique where the exit dose passes through the clinically unaffected eye.[24]

**Table 1.** *Breast cancer: radiation-induced sarcomas following radiotherapy*

| Cohort | No. of patients | Follow-up (years) | No. of sarcomas | | Relative risk | Incidence (%) | Reference |
|---|---|---|---|---|---|---|---|
| | | | Bone | ST | | | |
| San Francisco, University of California 1928–1958 | 445 | 5.0 | 1 | 0 | — | 0.22 | 11 |
| Boston, MA General Hospital 1953–1968 | 2 250 | 10.0 | 4 | 1 | — | 0.22 | 25 |
| Toronto, Princess Margaret Hospital 1958–1978 | 16 000 | 5.0 | 4 | 0 | — | 0.05 | 8 |
| University of Chicago 1927–1970 | 221 | 8–42 | 2 | 4 | 6.0 | 2.71 | 26 |
| Oslo, Norwegian Radium Hospital 1961–1971 | 2 764 | 10.0 | 2 | 1 | — | 0.27 | 13 |
| Marseille Cancer Institute* 1960–1981 | 2 280 | 7.9 | 0 | 2 | 3.4 | 0.02 | 27 |
| Duke University Medical Centre 1970–1981 | 140 | 6.3 | 0 | 0 | — | 0.00 | 28 |
| Villejuif, Institut Gustav Roussy 1954–1983 | 7 620 | 7.2 | 3 | 6** | 1.81 | 0.12 | 29 |
| Boston, Joint Center Radiation Therapy* 1968–1985 | 1 624 | 6.5 | 2 | 1 | — | 0.18 | 30 |
| Edinburgh, Western General Hospital 1954–1964 | 3 199 | > 18 | 4 | 1 | 7.3 (bone) NS (ST) | 0.26 | 31 |
| Milan, Istituto Tumori* 1973–1989 | 3 295 | 7.5 | 0 | 3 | 15.8 | 0.09 | 32 |
| West Sweden 1960–1980 | 13 490 | 8.4 | 0 | 12 | 2.2 | 0.09 | 33 |

* Breast conservation series.

**Excludes arm angiosarcomas (Stewart–Treves) syndrome.

NS = not significant; ST = soft tissue.

*Breast cancer*

Many cancer centres and registries have reported radiation-induced sarcomas in long-term survivors of breast cancer treated with radiotherapy,[8,11,13,25–33] and these are summarized in Table 1, which includes over 50 000 patients treated over the past 70 years with median follow-up of at least 5 years. The overall incidence for radiation-induced sarcomas was 0.10% (53/53 328). Most bone sarcomas arose in the scapula, humerus, clavicle or ribs and were osteosarcomas, and the majority were incurable.[34] Incidences were higher in series using orthovoltage radiotherapy than in later studies using megavoltage irradiation and, in the recent reports, soft tissue sarcomas outnumbered bone sarcomas. The highest incidence reported in a recent series was 1.10%, from 1382 consecutive autopsies on women dying with breast cancer (Roswell Park 1956–1988), but this is not comparable to the other reports because of selection factors.[35]

The large Swedish registry study of Karlsson *et al.*[33] reviewed in detail all cases of soft tissue sarcomas in 13 490 women treated for breast cancer over a 20-year period and found the mean annual incidence of soft tissue sarcomas was doubled to 0.02% compared to the normal population. One-third of their 18 cases had not received radiotherapy to the sarcoma site and, using a case-control analysis, half the radiation-induced sarcomas were found to have lymphoedema and/or high radiation doses as predisposing factors. The very low risk of sarcoma development, it was concluded, was likely to be even lower with the reduction in the dose and volume of breast irradiation and rarity of lymphoedema using the combined techniques of radiotherapy and surgery now widely adopted. Current standard practice consists of tangential beam irradiation with computer-assisted planning and it is uncommon to irradiate the axilla or supraclavicular fossa. Dose fractionation protocols have evolved to ensure good cosmesis is a major objective and bone and soft tissue complications are now rare, particularly at field junctions, which were a common site for sarcomas to arise.[29,30,36]

Many recent reports have described sarcomas arising in conserved breasts following the increased use of lumpectomy and breast irradiation as an alternative to mastectomy. The potential risk of radiation-induced sarcomas has been a controversial issue, and three cases have been reported in over

3000 patients followed prospectively since 1973, from the Milan Cancer Institute, the centre which reported the first randomized trial in breast conservation. Only one of the three cases was fatal. The rarity of these cases was such that no change in the current policy of conservative therapy with radiotherapy for breast cancer was advocated.[32] Angiosarcomas of the breast or overlying skin are now the most common sarcoma following breast conservation treatment,[37] and have a better prognosis than the other types of sarcoma, with most cases cured by mastectomy, particularly if they are diagnosed early. Malignant fibrous histiocytomas and fibrosarcomas have also been reported.[38]

Many case reports describe sarcomas which arose in areas of chronic radiation damage from excessive doses of orthovoltage radiotherapy, at junctions of adjacent radiation fields or at hot spots when current methods of computerized planning with tissue density corrections were not available. It is highly likely that such cases do not occur with modern radiotherapy practice, where a great deal of care is taken to avoid poor cosmetic results when breast conservation techniques for early cases of breast cancer are utilized as an alternative to mastectomy. The common sites of bone sarcomas, the scapula, humerus and clavicle, are usually omitted from current radiotherapy techniques, which do not routinely treat nodal regions.

### *Hodgkin's disease*

Patients with Hodgkin's disease are usually young and the vast majority are cured by radiotherapy and/or chemotherapy with few complications. MOPP (mustine, vincristine, procarbazine, prednisone) chemotherapy may induce acute nonlymphocytic leukaemia in the first decade of follow-up, but thereafter a growing number of patients are at risk of developing solid tumours, a small proportion of which are sarcomas induced by their therapeutic irradiation. Many cancer centres have reviewed their long-term survivors to report increasing rates of second malignancies with longer periods of follow-up, and collaborative groups have combined national and international cohorts of patients to assess the risks of sarcoma development, as detailed in Table 2. In 15 studies which followed a total of 69 000 patients with Hodgkin's disease treated since 1943, only 0.14% developed sarcomas following radiotherapy.[39–53] However, some reports do not provide sufficient details to determine whether all their cases would qualify as radiation-induced sarcomas. The most consistently reported solid tumours found following radiotherapy are non-Hodgkin's lymphoma, breast cancer and lung cancer.

Factors other than radiotherapy which are potentially related to the induction of these second malignancies include chemotherapy, which was given to the majority of patients, immunological abnormalities and a genetic predisposition, while other cases may be coincidental and found through the closer medical surveillance that occurs in young cancer survivors.

### *Testicular tumours*

Testicular cancers, like Hodgkin's disease, generally affect young adults and have cure rates exceeding 90%, with seminomas commonly treated by irradiation and teratomas usually receiving chemotherapy following orchidectomy. Many cancer institutions, national cancer registries and international cooperative groups have reviewed their long-term testicular cancer survivors and reported on second malignancies, and the larger studies are detailed in Table 3.[10,39,54–62] Some increases in the incidence of lung, gastrointestinal and genitourinary cancers have been found, but no study has found a significant risk of bone sarcomas, and the relative risk for soft tissue sarcomas varies from 1.0 to 5.4.[59] Of the 50 000 patients listed in the table, followed for a median duration of 5–15 years, 0.05% developed a subsequent sarcoma, and this figure includes some cases which may not have received radiotherapy or developed the sarcoma in tissues outside the radiation field.

Modifications to the treatment of testicular tumours in recent years include significant reductions in the dose and volume of radiotherapy, the omission of routine irradiation to the mediastinum, and the increasing use of surveillance and chemotherapy without irradiation, particularly in non-seminomas.[57] The addition of chemotherapy has been associated with some of the reported sarcomas, and there is a risk of acute leukaemia and bladder cancer following some cytotoxic regimes.[63] Combined treatment with radiotherapy and chemotherapy is rarely practised, unlike the situation with Hodgkin's disease where cure rates may be improved with optimum use of both modalities. Low-dose radiotherapy (16–20 Gy) is recommended for *in situ* carcinoma of the contralateral testis after positive biopsies, and can prevent the development of invasive germ cell tumours,[64] demonstrating the general acceptance that the benefits of irradiation far outweigh any risk of radiation-induced cancer.

Risk factors other than radiation have been identified in some case reports of radiation-induced sarcomas in testicular cancer patients, and it has been postulated that some teratomas transform into sarcomas spontaneously.[65,66] Both radiation and chemotherapy can produce differentiation of immature or undifferentiated teratomas.

### *Brain tumours*

Radiotherapy has been used in the treatment of benign brain tumours in large numbers of patients

**Table 2.** *Hodgkin's disease: sarcomas following radiotherapy*

| Cohort | No. of patients | Follow-up (years) | No. of sarcomas | | Relative risk | | Comments | Reference |
|---|---|---|---|---|---|---|---|---|
| | | | Bone | ST | Bone | ST | | |
| International Agency for Research on Cancer 1945–1984 | 28 462* | 4.1 | 5 | 4 | NS | NS | Sarcoma sites not identified | 39 |
| Seven published studies 1964–1981 | 6 513 | 5.4 | 4 | 11 | 10.0 | 10.0 | Sarcoma sites not identified | 40 |
| Italian centres 1969–1979 | 496 | 10.5 | 0 | 2 | NS | NS | Sarcoma sites not identified | 41 |
| International Database on Hodgkin's Disease 1960–1987 | 12 411* | 6.7 | 6 | 4 | 6.2 | NS | Sarcoma sites not identified | 42 |
| British National Lymphoma Investigation 1970–1987 | 1 859 | 6.5 | 2 | 0 | 15.0 | NS | Sarcoma sites not identified | 43 |
| Houston, MD Anderson Hospital 1966–1987 | 1 013* | >5.0 | 2 | 2 | NS | NS | Sarcoma sites not identified | 44 |
| Norwegian Radium Hospital 1968–1988 | 839 | 9.0 | 0 | 0 | NS | NS | One sarcoma outside radiation port | 45 |
| Florence, Italy 1960–1988 | 1 121* | >5.0 | 0 | 3 | NS | NS | Sarcoma sites not identified | 46 |
| Netherlands centres 1966–1986 | 1 761 | 9.2 | 0 | 3 | NS | 8.8 | All in radiation ports | 47 |
| North American centres 1940–1987 | 9 280* | 7.1 | 24 | | 0.9 | | Sarcoma sites not identified | 48 |
| Australasian Patterns of Care 1969–1988 | 820 | 10.0 | 2 | | NS | NS | Sarcoma sites not identified | 49 |
| Boston, JCRT 1969–1988 | 794 | 11.0 | 6 | | NS | NS | Sarcoma sites not identified | 50 |
| St Jude Children's Research Hospital 1962–1993 | 469 | 9.0 | 4 | 2 | NS | NS | Sarcoma sites not identified | 51 |
| Late Effects Study Group 1955–1986 | 1 270 | 11.4 | 4 | 2 | 24.6 | NS | Sarcoma sites not identified | 52 |
| Nordic countries 1943–1987 | 1 641* | 10.4 | 1 | 1 | NS | NS | One more sarcoma outside radiation port | 53 |

*Includes cases treated with chemotherapy alone.

who subsequently live out their full life span. Cases of radiation-induced sarcomas are rare and only 29 case reports of fibrosarcomas in the region of the pituitary fossa have been collected over the past 30 years in a literature review of irradiated pituitary tumours,[67] from an unknown patient denominator, often after excessively high doses. Only one sarcoma has been reported in five recent series of 1510 irradiated pituitary adenomas (0.07%) detailed in Table 4.[67–71] Some 47 cases of post-irradiation gliomas have also been reported,[69] but these too are rare and may be diminishing in frequency with modern radiotherapy techniques and equipment.

### *Head and neck cancers*

Excluding retinoblastomas, the risk of sarcomas following therapeutic irradiation in head and neck cancers is very low. In the 10 reports totalling 14 000 patients, summarized in Table 5,[12,72–80] the incidence was 0.16%. This is consistent with the one case of radiation-induced sarcoma for every 1250 treated patients estimated by Parsons in a

**Table 3.** *Testicular cancers: sarcomas following radiotherapy*

| Cohort | No. of patients | Follow-up (years) | No. of sarcomas | | Relative risk | | Comments | Reference |
|---|---|---|---|---|---|---|---|---|
| | | | Bone | ST | Bone | ST | | |
| Princess Margaret Hospital, Toronto | 652 | 9.1 | 1 | 0 | NS | NS | Incidence 0.15% | 10 |
| Scotland 1950–1969 | 547 | 15.4 | 0 | 0 | NS | NS | One sarcoma was outside the radiation portal | 54 |
| International Agency for Research on Cancer 1945–1984 | 17 730 | 6.4 | 1 | 7 | NS | 3.0 | Sarcoma sites not identified | 39 |
| South Thames Cancer Registry 1961–1980 | 1 004 | 6.8 | — | — | NS | NS | — | 55 |
| Norwegian Radium Hospital 1956–1977 | 876 | 12.7 | — | — | NS | NS | — | 56 |
| USA Patterns of Care 1973–1974 | 387 | 17.0 | 0 | 0 | NS | NS | — | 57 |
| Danish Cancer Registry 1943–1987 | 16 187 | 9.5 | 0 | 7 | NS | 2.4 | All sites irradiated | 58 |
| Netherlands Cancer Registry 1971–1985 | 1 909 | 7.7 | 1 | 2 | NS | 5.4 | Sarcoma sites not identified | 59 |
| Hannover 1970–1990 | 1 025 | 5.1 | 0 | 0 | NS | NS | One sarcoma occurred after chemotherapy | 60 |
| Berlin 1969–1992 | 584 | 6.0 | 0 | 0 | NS | NS | — | 61 |
| SEER Program, Connecticut Registry 1935–1991 | 9 739 | 7.0 | 0 | 6 | NS | 3.6 | Sarcoma sites not identified | 62 |

**Table 4.** *Pituitary adenomas: second malignancies following radiotherapy*

| Cohort | No. of patients | Follow-up (years) | No. of sarcomas | Other tumours | Relative risk | Reference |
|---|---|---|---|---|---|---|
| London, St Barts Hospital 1961–1982 | 332 | 11.0 | 0 | Glioma (2) Neuroblastoma (1) | NS | 67 |
| London, Royal Marsden Hospital 1962–1986 | 334 | 11.0 | Meningeal sarcoma (1) | Glioma (1) Meningioma (2) | 9.4 | 68 |
| Toronto, Princess Margaret Hospital 1972–1986 | 305 | 7.9 | 0 | Glioma (4) | 16 | 69 |
| Queensland Radium Institute 1962–1986 | 268 | 12.8 | 0 | — | NS | 70 |
| Edinburgh, Western General Hospital 1962–1990 | 271 | 8.0 | 0 | Lymphoma (1) | NS | 71 |

standard reference book,[81] assuming a 40% long-term survival rate. The most common second malignancies in patients irradiated for head and neck cancers are new carcinomas in the head and neck region, and lung and oesophageal carcinomas related to chronic exposure to the carcinogens of tobacco smoke and alcohol, compared to which radiation is a minor irritant.[77]

**Table 5.** *Head and neck cancers: radiation-induced sarcomas*

| Cohort | No. of patients | Follow-up (years) | No. of sarcomas | | Comments | Reference |
|---|---|---|---|---|---|---|
| | | | Bone | ST | | |
| Philadelphia, Fox Chase Center 1929–1973 | 611 | >5.0 | 1 | | Incidence 0.16% | 72 |
| Toronto, Princess Margaret Hospital 1958–1974 | 1 600 | >5.0 | 0 | 0 | — | 73 |
| Paris, Curie Institute 1957–1980 | 1 000 | >5.0 | 1 | 2 | Incidence 0.30% | 74 |
| Tokyo Medical and Dental University 1954–1985 | 1 429 | 4.6 | 0 | 1 | Incidence 0.07% | 75 |
| Los Angeles, University of California 1955–1979 | 2 151 | 5–30 | 0 | 0 | — | 76 |
| University of Rochester 1957–1983 | 235 | 10.0 | 0 | 0 | — | 77 |
| Netherlands Cancer Institute 1977–1993 | 2 500 | 2–17 | 0 | 5 | Incidence 0.20% | 78 |
| University of Florida 1964–1991 | 490 | 2–32 | 0 | 0 | — | 79 |
| National Taiwan University Hospital 1974–1988 | 2 112 | >5.0 | 8 | 0 | Incidence 0.38% | 12 |
| Chang Gung Hospital, Taipei 1979–1993 | 1 562 | 4.6 | 0 | 4 | Incidence 0.25% | 80 |

**Table 6.** *Cervical cancer: sarcomas following radiotherapy*

| Cohort | No. of patients | Follow-up (years) | No. of sarcomas | | Relative risk | | Comments | Reference |
|---|---|---|---|---|---|---|---|---|
| | | | Bone | ST | Bone | ST | | |
| Warsaw, Institute of Oncology 1948–1966 | 8 043 | 8.6 | 0 | 6 | NS | 2.9 | Incidence 0.07% (radiation induced) | 83 |
| International Radiation Study of Cervical Cancer 1960–1984 | 82 616 | 7.6 | 11 | 27 | NS | 1.9 | Sarcoma sites not identified | 82 |
| Danish Cancer Registry 1943–1982 | 20 727 | 10.1 | 5 | 26 | NS | 1.5 | Eleven cases in irradiated sites | 84 |
| Japanese institutions 1961–1981 | 7 694 | 7.1 | 0 | 2 | NS | NS | Both cases in uterus | 85 |
| International Cancer Registries Study 1935–1990 | 49 828 | 10.7 | 17 | 33 | 3.0 | 2.1 | Sarcoma sites not identified | 86 |

*Carcinoma of cervix*

Endocavitary and external beam radiotherapy have long been established as a standard method of treating cervical cancer. In 1985, an international collaboration involving 15 cancer registries in Europe and North America reported on the numbers of second cancers among a huge cohort of 182 000 women treated for cervical cancer, comparing those treated by radiotherapy and by surgery alone.[82] Connective tissue tumours were slightly increased in the irradiated group (relative risk 2.3 after 10 years), as were some other malignancies, including carcinomas of the bladder, rectum and other genital organs in heavily irradiated tissues, but not bone tumours. Sarcoma sites were not identified and the number occurring away from the radiation port was not stated. Overall, the number of cancers which could be attributed to irradiation was estimated to be at most 5%, and this was more than counterbalanced by the reduction in the incidence of breast cancer, probably secondary to ovarian ablation. Three of four smaller studies showed similar trends.[83–86] Table 6 shows the reported cases of sarcomas in 170 000 long-term survivors of radiotherapy, for an overall incidence of 0.08%.

In a follow-up study from the International Radiation Study of Cervical Cancer, Boice *et al.* analyzed 38 sarcomas according to site using case-matched controls and found no significant increase in the risk for bone sarcomas but some increased risk for soft tissue sarcomas (both relative risks 1.9) which occurred within the irradiated region.[87] The risk of pelvic soft tissue sarcomas was doubled following cervical radiotherapy. Despite many anecdotal reports of uterine tumours including sarcomas occurring after radiotherapy,[88,89] a strong body of epidemiological literature has not identified a definite relationship between radiotherapy and sarcomas arising from the uterus.[90]

### Conclusions

Radiotherapy has an established place in the treatment of a wide range of neoplastic conditions, and the proportion of cured patients continues to grow. Virtually all will enjoy a further 10 years of life before they approach the time at which any sarcoma induced by their earlier therapy could appear. Despite numerous anecdotal reports of sarcomas arising in cancer patients who have received radiotherapy, large epidemiological studies involving hundreds of thousands of radiotherapy patients have shown that at most only 5% of all second primary cancers can be convincingly linked to the radiation treatment,[91] and all the others are attributable to life-style, inheritance and other carcinogens. Of the 360 000 radiotherapy patients evaluated in the reports in this review, only 0.1% or one patient in a thousand has subsequently developed sarcomas which meet the criteria of being radiation induced.

The risk of sarcoma development following irradiation is extremely low and should be weighed against the risks of death and carcinogenicity from hormonal anti-cancer agents (e.g. cardiovascular toxicity and vaginal adenocarcinoma from stilboestrol, breast cancer from oestrogens, endometrial cancer from tamoxifen) or chemotherapeutic drugs (e.g. leukaemia from alkylating agents and etoposide, bladder cancer and possibly sarcoma from cyclophosphamide[92]), or the operative morbidity and mortality of surgery, all alternatives to radiotherapy in the treatment of cancer patients.[15] Potential carcinogenicity should not be regarded as a contraindication to the use of radiotherapy, even in patients with retinoblastoma and neurofibromatosis where a higher risk of tumour induction is recognized. In the more common cancers as well, the long-term benefit derived from radiotherapy far outweighs the serious side-effects, provided standard techniques are used.

Radiation-induced sarcomas often arise in areas heavily damaged by radiation doses far in excess of normal tissue tolerance and they are likely to be seen even less frequently with current standards of treatment which use lower doses of megavoltage irradiation delivered in daily fractions of 2 Gy, avoid multiple courses of treatment and utilize planning by modern computer-assisted techniques, resulting in acceptable acute toxicity and infrequent chronic bone and soft tissue complications. Another much-feared potential complication, the anaplastic transformation of benign or low-grade tumours following irradiation, occurs even less frequently than radiation-induced sarcomas, and cases identical to those anecdotally attributed to irradiation have also been found following other types of treatment in the absence of radiotherapy.[93–95]

There is evidence in recent reports of radiation-induced sarcomas that aggressive management with early diagnosis and complete local resection results in prolonged survival no different from sarcomas unrelated to prior irradiation, particularly if they develop in soft tissue rather than bone.[23,30,36–38,96] Regular follow-up examinations of cancer patients at the centre where their treatment took place should promote early detection by prompt investigation of any new masses, with biopsies to exclude the alternative diagnosis of relapse from the original cancer which commonly delays optimal sarcoma treatment. Complete excisions should be performed and consideration given to post-operative re-irradiation as occurs for primary soft tissue sarcomas. A role for chemotherapy in treatment is yet to be established, and some agents may increase the incidence of these rare tumours.[21,91]

### References

1 Frieben A. Cancroid des rechten Handrükens, das sich nach langdavernder Einwirkung von Roentgenstrahlen. *Fortchr Röntgenstr* 1902; 6:106.
2 Perthes G. Zur Frage der Röntgentherapie des Carcinoms. *Arch Klin Chirurg* 1904; 74:400–5.
3 Beck A. Zur frage des Röntgensarkoms, zugleich ein Beitrag zur Pathogenese des Sarkoms. *München Medizin Wochenschr* 1922; 69:623–4.
4 Martland HS. Occupational poisoning in manufacture of luminous watch dials. *J Am Med Assoc* 1929; 92:466–73.
5 Cahan WG, Woodard HQ, Higinbotham NL, *et al.* Sarcoma arising in irradiated bone. Report of eleven cases. *Cancer* 1948; 1:3–29.
6 Robinson E, Neugut AI, Wylie P. Clinical aspects of postirradiation sarcomas. *J Natl Cancer Inst* 1988; 80:233–40.
7 Wiklund TA, Blomqvist CP, Räty J, *et al.* Postirradiation sarcoma. Analysis of a nationwide cancer registry material. *Cancer* 1991; 68:524–31.
8 Tountas AA, Fornasier VL, Harwood AR, *et al.* Postirradiation sarcoma of bone. A perspective. *Cancer* 1979; 43:182–7.
9 Tucker MA, D'Angio GJ, Boice JD, *et al.* Bone sarcomas linked to radiotherapy and chemotherapy in children. *N Engl J Med* 1987; 317:588–93.
10 Harwood AR, Yaffe M. Cancer in man after diagnostic or therapeutic irradiation. *Cancer Surv* 1982; 1:703–31.
11 Phillips TL, Sheline GE. Bone sarcomas following radiation therapy. *Radiology* 1963; 81:992–6.
12 Ko JY, Chen CL, Lui LT, *et al.* Radiation-induced malignant fibrous histiocytoma in patients with nasopharyngeal carcinoma. *Arch Otolaryngol Head Neck Surg* 1996; 122:535–8.
13 Hatlinghus S, Rode L, Christensen I *et al.* Sarcoma following irradiation for breast cancer. Report of three unusual cases including one malignant mesenchymoma of bone. *Acta Radiol Oncol* 1986; 25:239–42.
14 Brady MS, Gaynor JJ, Brennan MF. Radiation-associated sarcoma of bone and soft tissue. *Arch Surg* 1992; 127:1379–85.
15 Mark RJ, Poen J, Tran LM, *et al.* Postirradiation sarcomas. A single-institution study and review of the literature. *Cancer* 1994; 73:2653–62.
16 Abramson DH, Ellsworth RM, Kitchin D, *et al.* Second nonocular tumors in retinoblastoma survivors. Are they radiation-induced? *Ophthalmology* 1984; 91:1351–5.
17 Ducatman BS, Scheithauer BW. Postirradiation neurofibrosarcoma. *Cancer* 1983; 51:1028–33.
18 Garber JE, Goldstein AM, Kantor AF, *et al.* Follow-up study of twenty-four families with Li-Fraumeni syndrome. *Cancer Res* 1991; 51:6094–7.
19 Lavey RS, Eby NL, Prosnitz LR. Impact of second malignancy risk of the combined use of radiation and chemotherapy for lymphomas. *Cancer* 1990; 66:80–8.
20 Tarbell NJ, Gelber RD, Weinstein HJ, *et al.* Sex differences in risk of second malignant tumours after Hodgkin's disease in childhood. *Lancet* 1993; 341:1428–32.
21 Heyn R, Haeberlen V, Newton WA, *et al.* Second malignant neoplasms in children treated for rhabdomyosarcoma. *J Clin Oncol* 1993; 11:262–70.
22 Eng C, Li FP, Abramson DH, *et al.* Mortality from second tumors among long-term survivors of retinoblastoma. *J Natl Cancer Inst* 1993; 85:1121–8.
23 Smith LM, Donaldson SS, Egbert PR, *et al.* Aggressive management of second primary tumors in survivors of hereditary retinoblastoma. *Int J Radiat Oncol Biol Phys* 1989; 17:499–505.
24 Plowman PN, Kingston JE, Hungerford JL. Prophylactic retinal radiotherapy has an exceptional place in the management of familial retinoblastoma. *Br J Cancer* 1993; 68:743–5.
25 Hatfield PM, Schulz MD. Postirradiation sarcoma. Including 5 cases after X-ray therapy of breast cancer. *Radiology* 1970; 96:593–602.
26 Ferguson DJ, Sutton HG, Dawson PJ. Late effects of adjuvant radiotherapy for breast cancer. *Cancer* 1984; 54:2319–23.
27 Kurtz JM, Amalric R, Brandone H, *et al.* Contralateral breast cancer and other second malignancies in patients treated by breast-conserving therapy with radiation. *Int J Radiat Oncol Biol Phys* 1988; 15:277–84.
28 Lavey RS, Eby NL, Prosnitz LR. Impact of radiation therapy and/or chemotherapy on the risk for a second malignancy after breast cancer. *Cancer* 1990; 66:874–81.
29 Taghian A, De Vathaire F, Terrier P, *et al.* Long-term risk of sarcoma following radiation treatment for breast cancer. *Int J Radiat Oncol Biol Phys* 1991; 21:361–7.
30 Pierce SM, Recht A, Lingos TI, *et al.* Long-term radiation complications following conservative surgery (CS) and radiation therapy (RT) in patients with early stage breast cancer. *Int Radiat Oncol Biol Phys* 1992; 23:915–23.
31 Doherty MA, Rodger A, Langlands AO, *et al.* Multiple primary tumours in patients treated with radiotherapy for breast cancer. *Radiother Oncol* 1993; 26:125–31.
32 Zucali R, Merson M, Placucci M, *et al.* Soft tissue sarcoma of the breast after conservative surgery and irradiation for early mammary cancer. *Radiother Oncol* 1994; 30:271–3.
33 Karlsson P, Holmberg E, Johansson KA, *et al.* Soft tissue sarcoma after treatment for breast cancer. *Radiother Oncol* 1996; 38:25–31.
34 Doherty MA, Rodger A, Langlands AO. Sarcoma of bone following therapeutic irradiation for breast carcinoma. *Int J Radiat Oncol Biol Phys* 1986; 12:103–6.
35 Mamounas EP, Perez-Mesa C, Penetrante RB, *et al.* Patterns of occurrence of second primary non-mammary malignancies in breast cancer patients: results from 1382 consecutive autopsies. *Surg Oncol* 1993; 2:175–85.
36 Bobin JY, Rivoire M, Delay E, *et al.* Radiation induced sarcomas following treatment for breast cancer: presentation of a series of 14 cases treated with an aggressive surgical approach. *J Surg Oncol* 1994; 57:171–7.
37 Cafiero F, Gipponi M, Peressini A, *et al.* Radiation-associated angiosarcoma. Diagnostic and therapeutic implications—two case reports and a review of the literature. *Cancer* 1996; 77:2496–502.
38 Lemson MS, Mud HJ, Wijnmaalen AJ, *et al.* Post-radiation fibrosarcoma of the breast. *The Breast* 1996; 5:368–71.
39 Kaldor JM, Day NE, Band P, *et al.* Second malignancies following testicular cancer, ovarian cancer and Hodgkin's disease: an international collaborative study among cancer registries. *Int J Cancer* 1987; 39:571–85.
40 Boivin JF, O'Brien K. Solid cancer risk after treatment of Hodgkin's disease. *Cancer* 1988; 61:2541–6.
41 Cimino G, Papa G, Tura S, *et al.* Second primary cancer following Hodgkin's disease: updated results of an Italian multicentric study. *J Clin Oncol* 1991; 9:432–7.
42 Henry-Amar M. Second cancer risk after the treatment for Hodgkin's disease: a report from the International Database on Hodgkin's Disease. *Ann Oncol* 1992; 3:S117–28.
43 Swerdlow AJ, Douglas AJ, Vaughan Hudson G, *et al.* Risk of second primary cancers after Hodgkin's disease by type of treatment: analysis of 2846 patients in the British National Lymphoma Investigation. *Br Med J* 1992; 304:1137–43.
44 Rodriguez MA, Fuller LM, Zimmerman SO, *et al.* Hodgkin's disease: study of treatment intensities and incidences of second malignancies. *Ann Oncol* 1993; 4:125–31.
45 Biti G, Cellai E, Magrini SM, *et al.* Second solid tumors and leukemia after treatment for Hodgkin's disease: an analysis of 1121 patients from a single institution. *Int J Radiat Oncol Biol Phys* 1994; 29:25–31.
46 Abrahamsen JF, Andersen A, Hannisdal E, *et al.* Second malignancies after treatment of Hodgkin's disease: the influence of treatment, follow-up time and age. *J Clin Oncol* 1993; 11:255–61.
47 van Leeuwen FE, Klokman WJ, Hagenbeek A, *et al.* Second cancer risk following Hodgkin's disease: a 20-year follow-up study. *J Clin Oncol* 1994; 12:312–25.
48 Boivin JF, Hutchison GB, Zauber AG, *et al.* Incidence of second cancers in patients treated for Hodgkin's disease. *J Natl Cancer Inst* 1995; 87:732–41.
49 Barton M, Boyages J, Crennan E, *et al.* Radiation therapy for early stage Hodgkin's disease: Australasian Patterns of Care. *Int J Radiat Oncol Biol Phys* 1995; 31:227–36.
50 Mauch PM, Kalish LA, Marcus KC, *et al.* Long-term survival in Hodgkin's disease. Relative impact of mortality, second tumors, infection, and cardiovascular disease. *Cancer J Sci Am* 1995; 1:33–42.
51 Beaty O, Hudson MM, Greenwald C, *et al.* Subsequent malignancies in children and adolescents after treatment for Hodgkin's disease. *J Clin Oncol* 1995; 13:603–9.
52 Bhatia S, Robison LL, Oberlin O, *et al.* Breast cancer and other second neoplasms after childhood Hodgkin's disease. *N Engl J Med* 1996; 334:745–51.
53 Sankila R, Garwicz S, Olsen JH, *et al.* Risk of subsequent malignant neoplasms among 1641 Hodgkin's disease patients diagnosed in childhood and adolescence: a population-based cohort study in the five Nordic countries. *J Clin Oncol* 1996; 14:1442–6.
54 Hay JH, Duncan W, Kerr GR. Subsequent malignancies in patients irradiated for testicular tumours. *Br J Radiol* 1984; 57:597–602.
55 Coleman MP, Bell CMJ, Fraser P. Second primary malignancy after Hodgkin's disease, ovarian cancer and cancer of the testis: a population-based cohort study. *Br J Cancer* 1987; 56:349–55.
56 Fossa SD, Langmark F, Aass N, Andersen A, *et al.* Second non-germ cell malignancies after radiotherapy of testicular cancer with or without chemotherapy. *Br J Cancer* 1990; 61:639–43.
57 Hanks GE, Peters T, Owen J. Seminoma of the testis: long term beneficial and deleterious results of radiation. *Int J Radiat Oncol Biol Phys* 1992; 24:913–19.
58 Jacobsen GK, Mellemgaard A, Engelholm SA, *et al.* Increased incidence of sarcoma in patients treated for testicular seminoma. *Eur J Cancer* 1993; 29A:664–8.
59 van Leeuwen FE, Stiggelbout AM, van den Belt-Dusebout AW, *et al.* Second cancer risk following testicular cancer: a follow-up study of 1909 patients. *J Clin Oncol* 1993; 11:415–24.
60 Bokemeyer C, Schmoll HJ. Secondary neoplasms following treatment of malignant germ cell tumors. *J Clin Oncol* 1993; 11:1703–9.
61 Dieckmann KP, Wegner HEH, Krain J. Multiple primary neoplasms in patients with testicular germ cell tumor. *Oncology* 1994; 51:450–8.
62 Travis LB, Curtis RE, Hankey BF. Correspondence: Second malignancies after testicular cancer. *J Clin Oncol* 1995; 13:533–4.
63 Bokemeyer C, Schmoll HJ. Treatment of testicular cancer and the development of secondary malignancies. *J Clin Oncol* 1995; 13:283–92.
64 Giwercman A, von der Maase H, Berthelsen JG, *et al.* Localized irradiation of testes with carcinoma *in situ*: effects on Leydig cell function and eradication of malignant germ cells in 20 patients. *J Clin Endocrinol Metab* 1991; 73:596–603.
65 Radford DM, Johnson FE, Janney CG. Sarcomatous change in a teratoma after treatment of testicular carcinoma. *Cancer* 1991; 68:395–9.
66 Lee KC, Yeung K, Welsh C, *et al.* Angiosarcoma following treatment of testicular seminoma: case report and literature review. *J Urol* 1995; 153:1055–6.
67 Jones A. Radiation oncogenesis in relation to the treatment of pituitary tumours. *Clin Endocrinol* 1991; 35:379–97.
68 Brada M, Ford D, Ashley S, *et al.* Risk of second brain tumour after conservative surgery and radiotherapy for pituitary adenoma. *Br J Med* 1992; 304:1343–6.
69 Tsang RW, Laperriere NJ, Simpson WJ, *et al.* Glioma arising after radiation therapy for pituitary adenoma. A report of four patients and estimation of risk. *Cancer* 1993; 72:2227–33.
70 Hughes MH, Llamas KJ, Yelland ME, *et al.* Pituitary adenomas: long-term results for radiotherapy alone and post-operative radiotherapy. *Int J Radiat Oncol Biol Phys* 1993; 27:1035–43.
71 Bliss P, Kerr GR, Gregor A. Incidence of second brain tumours after pituitary irradiation in Edinburgh 1962–1990. *Clin Oncol* 1994; 6:361–3.
72 Seydel HG. The risk of tumor induction in man following medical irradiation for malignant neoplasm. *Cancer* 1975; 35:1641–5.
73 Brown M. Second primaries in cases of cancer of the larynx. *J Laryngol Otol* 1978; 92:991–6.
74 Steeves RA, Bataini JP. Neoplasms induced by megavoltage radiation in the head and neck region. *Cancer* 1981; 47:1770–4.
75 Shibuya H, Hisamitsu S, Shioiri S, *et al.* Multiple primary cancer risk in patients with squamous cell cancer of the oral cavity. *Cancer* 1987; 60:3083–6.
76 Parker RG, Enstrom JE. Second primary cancers of the head and neck following treatment of initial primary head and neck cancers. *Int J Radiat Oncol Biol Phys* 1988; 14:561–4.
77 McDonald S, Haie C, Rubin P, *et al.* Second malignant tumors in patients with laryngeal carcinoma: diagnosis, treatment and prevention. *Int J Radiat Oncol Biol Phys* 1989; 17:457–65.
78 van der Laan BFAM, Baris G, Gregor RT, *et al.* Radiation-induced tumours of the head and neck. *J Laryngol Otol* 1995; 109:346–9.
79 Fein DA, Lee WR, Amos WR, *et al.* Oropharyngeal carcinoma treated with radiotherapy: a 30-year experience. *Int J Radiat Oncol Biol Phys* 1996; 34:289–96.
80 Wang CC, Hong JH, Hsu KH, *et al.* Second malignant tumors in Chinese patients with nasopharyngeal carcinoma following radiotherapy. *Int J Radiat Oncol Biol Phys* 1996; 36 Suppl:163 (abstract).
81 Parsons JT. The effect of radiation on normal tissues of the head and neck. In: Million RR, Cassisi NJ (eds). *Management of head and neck cancer. A multidisciplinary approach*, 2nd edn. Philadelphia: JB Lippincott, 1994:284.
82 Boice JD, Day NE, Andersen A, *et al.* Second cancers following radiation treatment for cervical cancer. An international collaboration among cancer registries. *J Natl Cancer Inst* 1985; 74:955–75.
83 Czesnin K, Wronkowski Z. Second malignancies in the irradiated area in patients treated for uterine cervix cancer. *Gynecol Oncol* 1978; 6:309–15.
84 Storm HH. Second primary cancer after treatment for cervical cancer. Late effects after radiotherapy. *Cancer* 1988; 61:679–88.
85 Arai T, Nakano T, Fukuhisa K, *et al.* Second cancer after radiation therapy for cancer of the uterine cervix. *Cancer* 1991; 67:398–405.
86 Kleinerman RA, Boice JD, Storm HH, *et al.* Second primary cancer after treatment for cervical cancer. An International Cancer Registries Study. *Cancer* 1995; 76:442–52.
87 Boice JD, Engholm G, Kleinerman RA, *et al.* Radiation dose and second cancer risk in patients treated for cancer of the cervix. *Radiat Res* 1988; 116:3–55.
88 Chumas JC, Patsner B, Mann WJ. High-grade pelvic sarcoma after radiation therapy for low-grade endometrial stromal sarcoma. *Gynecol Oncol* 1990; 36:428–31.
89 Parkash V, Carcangiu ML. Uterine papillary serous carcinoma after radiation therapy for carcinoma of the cervix. *Cancer* 1992; 69:496–501.
90 Mettler FA, Upton AC. Carcinogenesis of specific organ sites. In: *Medical effects of ionizing radiation*, 2nd edn. Philadelphia: WB Saunders, 1995:164–6.
91 Hall EJ. Radiation carcinogenesis. In: *Radiobiology for the radiologist*, 4th edn. Philadelphia: JB Lippincott, 1994; 336.
92 Pedersen-Bjergaard J, Jonsson V, Pedersen M, *et al.*

Correspondence. Leiomyosarcoma of the urinary bladder after cyclophosphamide. *J Clin Oncol* 1995; 13:532–3.
93 Makek MS, Andrews JC, Fisch U. Malignant transformation of a nasopharyngeal angiofibroma. *Laryngoscope* 1989; 99:1088–92.
94 Hefti FL, Gaechter A, Remagen W, *et al.* Recurrent giant-cell tumor with metaplasia and malignant change, not associated with radiotherapy. *J Bone Joint Surg* 1992; 74A:930–4.
95 Tharp ME, Shidnia H. Radiotherapy in the treatment of verrucous carcinoma of the head and neck. *Laryngoscope* 1995; 105:391–6.
96 Bloehle C, Peiper M, Schwarz R, *et al.* Post-irradiation soft tissue sarcoma. *Eur J Cancer* 1995; 31A:31–4.
